# Birth preparedness and complication readiness among pregnant women in Osogbo Metropolis, Southwest Nigeria

**DOI:** 10.11604/pamj.2017.27.74.7266

**Published:** 2017-06-01

**Authors:** Adedayo Olukemi Sabageh, Oluwatosin Adediran Adeoye, Adeleye Abiodun Adeomi, Donatus Sabageh, Adebola Afolake Adejimi

**Affiliations:** 1 Department of Community Medicine, Ladoke Akintola University of Technology, Ogbomoso, Oyo State, Nigeria; 2 Department of Community Medicine, Ladoke Akintola University of Technology Teaching Hospital, Ogbomoso, Oyo State, Nigeria; 3 Department of Morbid Anatomy and Histopathology, Ladoke Akintola University of Technology, Ogbomoso, Oyo State, Nigeria

**Keywords:** Birth preparedness, complication readiness, pregnancy, delivery, women, Nigeria

## Abstract

**Introduction:**

High maternal mortality is a major problem in Nigeria. Birth Preparedness and Complication Readiness will ensure that women can have professional delivery thus reducing obstetric complications. This study assessed the birth preparedness and complication readiness among pregnant women in Osogbo metropolis, a south western community in Nigeria.

**Methods:**

A community based descriptive cross sectional survey was used. A total of 180 women were selected using multistage sampling technique. Pretested semi-structured interviewer administered questionnaires were used to elicit information about previous obstetric history, knowledge of the danger signs of pregnancy and level of birth preparedness. Composite score and mean were computed. Data analysis was done using Statistical Package for Social Sciences (SPSS) version 17. P-value was set at < 0.05.

**Results:**

The mean age was 26.11 ± 3.63 years. A total of 51.1% were carrying their 2^nd^ or 3^rd^pregnancies. A total of 70.8% were aware of danger signs in pregnancy and the commonest danger sign mentioned was bleeding per vagina. In all, 82.1% were well prepared for birth. Being in the younger age group (p = 0.026), being more educated (p < 0.0001) and being aware of danger signs in pregnancy (p < 0.0001) was more significantly associated with being well prepared.

**Conclusion:**

The respondents were well prepared for birth with the younger women, educated ones and those knowledgeable of danger signs being better prepared. Continuous education about the Birth Preparedness and Complication Readiness should be sustained in order to maintain and improve women's preparedness.

## Introduction

High maternal mortality is a major problem in the developing world [1, 2], and Nigeria is no exception [3, 4]. Beyond the maternal death, there may also be associated perinatal and neonatal death, and these indices too has not improved significantly in Nigeria [3, 4]. All these are due mainly to the 3 phases of delay usually experienced in maternal care [5, 6] which stems from inadequate or outright lack of birth and emergency preparedness. Birth preparedness helps ensure that women can reach professional delivery care when labor begins and reduces the delays that occur when women experience obstetric complications [1, 7]. It ensures the readiness and timely utilization of skilled maternal and neonatal health care. irth Preparedness and Complication Readiness (BPACR) is a safe motherhood initiative with the objective of planning for normal birth and anticipating the actions needed in case of an emergency [1, 7]. Pregnant women, their families, and communities are encouraged to effectively plan for births and deal with any anticipated emergencies, if they occur [8]. The major components of BPACR involved knowledge of danger signs, identification of a skilled birth attendant; identification of the closest appropriate care facility, plan for transportation to this care facility for delivery and/or obstetric emergencies, save money to pay for care and other resources, identification of a potential blood donor and decision maker in case of emergency [1, 5, 7, 8]. Other elements of birth preparedness include knowledge of expected date of delivery and signs of labour, HIV testing, arranging for someone to take care of the family during delivery, importance of postnatal care, importance of exclusive breastfeeding and contraception [7, 9]. In spite of the benefit of BPACR in the reduction of the 3 phases of delay and thus reduction of maternal as well as neonatal deaths and complications, [10, 11] studies in the developing countries have not shown adequate preparedness [5, 8, 12]. In India, 47.8% of mothers were found to be well-prepared, and the remaining 52.2% were less-prepared [8]. Only a quarter of pregnant women in an Ethiopian study identified a skilled provider for her care, just 8.1% identified health facility for delivery and/or for obstetric emergencies and planned to deliver at health facilities, as low as 2.3% identified potential blood donor in case of emergency. Generally, just 17% of the pregnant women in the study were considered as well prepared for birth and complications [1]. Study of BPACR among pregnant women in an Ante Natal Care (ANC) clinic in Kenya revealed that a significant 44.9% of the respondents had not made prior transport arrangements to get to hospital in case of an emergency while 37.1% had not set aside funds for emergency purposes [9]. Iliyasu et al in their study on Birth Preparedness, Complication Readiness and Fathers' Participation in Maternity Care in a Northern Nigerian Community found out that majority of pregnancies were unplanned while most men (71.5%) made plans for the baby's naming ceremony, less than a third made plans for mother's health care, transportation and delivery and only 19.5% made savings for obstetric emergencies; decisions on place of delivery, arrangement for skilled assistance at delivery and preparations for blood donation were found to be made by only 9.0%, 6.2% and 0.8% of respondents respectively [5]. The presence of skilled attendants at births and availability of emergency obstetric care have been shown to greatly reduce maternal deaths due to obstetric complications [13–15] and women who were well prepared for childbirth were more likely to choose assistance by skilled birth attendants (SBA) than those who were not well prepared [11]. It is thus important to examine the level of birth preparedness among Nigerian women especially as statistics have shown that only 38% of births were being attended to by SBA in Nigeria [4]. This study therefore assessed the birth preparedness and complication readiness among pregnant women in Osogbo metropolis, a south western community in Nigeria.

## Methods

The study was a community based descriptive cross sectional survey carried out in Osogbo which is the capital of Osun State, South-western, Nigeria. Osogbo is made up of two local governments; they are Olorunda local government and Osogbo local government. One hundred and eighty women were selected for interview using multi stage sampling technique. In the first stage, 3 wards were randomly selected from each of the two local governments in the metropolis to make six. Equal probability technique was used to allocate 30 questionnaires to each selected ward, and houses in the wards were visited for the administration of the questionnaires to eligible respondents until they were exhausted. In houses where there were more than one eligible respondent, one respondent was selected by balloting for the interview. A pretested semi-structured interviewer administered questionnaire was used to elicit information. The questionnaire inquired about respondents' demographic characteristics, previous obstetric history and knowledge about danger signs of pregnancy and delivery. The main components of BPACR were also asked in the fourth section of the questionnaire to assess respondents' level of birth preparedness. The main questions were on identification of place of delivery, SBA attendant present at place of delivery, transportation arrangement made, money saved for delivery and blood donor arrangement made; each ''Yes'' answer was awarded 1 mark, and 0 was given for ''No'' answers (except for the second question on SBA where Yes was 2, I do not know was 1 and No was 0). Composite score and mean was thereafter computed and respondents that score below the mean were classified as poorly prepared while those that had the mean score and above were taken as well prepared. Data analysis was done using Statistical Package for Social Sciences (SPSS) version 17. Variables were expressed in frequency tables and pie chart. Pearson's chi square was used in the association of respondents' socio-demographic factors and awareness of danger signs in pregnancy with level of birth preparedness. Level of statistical significance was set at p-value < 0.05. Ethical approval was received from each of the local government authorities and verbal consent was obtained from the respondents after explaining the objectives of the study to them.

## Results

The result showed majority of the respondents (81 (45.0%)) to be in the age group 21-25 years, followed by the age group 26-30 years 65 (36.1%). The mean age was 26.11 ± 3.63 years. They were predominantly from the urban areas of the town (113 (62.8%)) and 94 (52.2%) were Muslims; 822 (45.6%) were Christians while the remaining 4 (2.2%) practiced traditional religion. A hundred and fifty eight (87.8%) were Yorubas, 13 (7.2%) were Ibos, 6 (3.3%) were Hausas and the remaining 3 (1.7) were from other tribes. Majority (96 (53.3%)) had completed secondary school and (87 (48.3%)) were skilled workers (**Table 1**). **Table 2** showed the obstetric history of respondents and 92 (51.1%) were carrying their 2nd or 3rd pregnancies while 62 (34.5) were primigravida. Among the 118 that were not primigravida, 67 (56.8%) had one child alive, 111 (94.4%) said they attended ANC in their last pregnancy, 91 (76.8%) had no complications, 98 (83.1) delivered in a health care facility and 93 (79.2%) had no complication during delivery. Spontaneous vaginal delivery was the commonest method of delivery as reported by 103 (87.4%) respondents, 14 (11.8%) had instrumental delivery, while 1 (0.8%) person had caesarean section. When asked about the danger signs of pregnancy, 127 (70.8%) were aware of danger signs in pregnancy and the commonest danger sign mentioned by 71 (55.9) of the 127 was bleeding per vagina, other danger signs mentioned were leg swelling 9 (7.1%), headache 15 (11.8%), body pain 32 (25.2%), dizziness (20 (15.7%)) and fainting attack (6 (4.7%)). A lesser proportion of 88 (49.0%) respondents were however found to be aware of complications during delivery, amongst which bleeding per vagina was also the commonest complication mentioned by 30 (34.1%) respondents. Majority have identified place of delivery (170 (94.4%)), with SBA in place (142 (83.5%)), made transportation arrangement in advance (152 (89.3%)), were saving money towards the delivery (166 (92.4%))and for possible caesarean section (142 (78.8%)). A hundred and seventy four (96.5%) had the essential baby needs ready, 109 (60.8%) had made arrangement for blood donor and 170 (94.3%) made plans for baby's special care if needed. Assessing the general level of birth preparedness showed 148 (82.1%) to be well prepared while the remaining 32 (17.9%) were not well prepared. **Table 3** shows the association of some variables with the level of preparedness of respondents. Being in the younger age group (p = 0.026), being a Ibo (p < 0.0001) being more educated (p < 0.0001) and being aware of danger signs in pregnancy (p < 0.0001) was more significantly associated with being well prepared for birth.

**Table 1:** Socio demographic characteristics of respondents

Variable	Frequency (n=180)	Percent (%)
**Age in years**		
≥20	7	3.9
21 – 25	81	45.1
26 – 30	65	36.1
31 – 35	27	15.0
**Location settings**		
Urban	113	62.8
Rural	67	37.2
**Religion**		
Christianity	82	45.6
Islam	94	52.2
Traditional beliefs	4	2.2
**Tribe**		
Yoruba	158	87.8
Ibo	13	7.2
Hausa/Fulani	6	3.3
Others	3	1.7
**Highest level of education**		
No formal education	4	2.2
Primary education	24	13.3
Secondary education	96	53.3
Tertiary/Postgraduate education	56	31.1
**Occupation**		
Unemployed	45	25.0
Unskilled	11	6.1
Skilled	87	48.3
Professionals	37	20.6

**Table 2:** Respondents’ obstetric history

Variables	Frequency	Percent (%)
**Numbers of pregnancy (including index pregnancy) (n=180)**		
1	62	34.5
2-3	92	51.1
4-5	26	14.4
**Had ANC care in last pregnancy (n=118)**		
No	7	5.6
Yes	111	94.4
**Had complications in last pregnancy (n=118)**		
Yes	27	23.2
No	91	76.8
**Complications experienced in last pregnancy (n=27)**		
Leg swelling	9	31.6
Malaria	5	21.0
Back/abdominal pain	4	15.8
Vomiting and dizziness	9	31.6
**Delivery centre in last pregnancy (n=118)**		
Health facility/Hospital	98	83.1
Mission homes	14	11.9
TBA’s place	6	5.1
**Delivery method of last pregnancy (n=118)**		
Vaginal	103	87.4
Instrumental	14	11.8
Caesarean section	1	0.8
**Had complications during last delivery (n=118)**		
Yes	25	20.8
No	93	79.2
**Complication experienced (n=25)**		
Abnormal lie	3	10.5
Bleeding	4	15.8
Fever	5	21.1
Prolonged Labour	13	52.6

**Table 3:** Association of some variables with respondents’ level of birth preparedness

Variables	Well Prepared (n=148)	Poorly Prepared (n=32)	χ^2^	p-value
**Age**				
≥20	7 (100.0)	0 (0.0)	9.233**	0.026*
21 – 25	60 (74.1)	21 (25.9)		
26 – 30	56 (86.7)	9 (13.3)		
31 – 35	25 (92.3)	2 (7.7)		
**Location settings**				
Urban	96 (85.0)	17 (15.0)	1.552	0.213
Rural	52 (77.6)	15 (22.4)		
**Tribe**				
Yoruba	134 (84.8)	24 (15.2)	22.708**	<0.0001*
Ibo	11 (85.7)	2 (14.3)		
Hausa/Fulani	0 (0.0)	6 (100.0)		
Others	3 (100.0)	0 (0.0)		
**Highest level of education**				
None	0 (0.0)	4 (100.0)	22.453**	<0.0001*
Primary	15 (62.5)	9 (37.5)		
Secondary	83 (86.0)	13 (14.0)		
Tertiary/Postgraduate	50 (88.6)	6 (11.4)		
**Occupation**				
Unemployed	36 (81.0)	9 (19.0)	5.141	0.162
Unskilled	7 (66.7)	4 (33.3)		
Skilled	71 (82.1)	16 (17.9)		
Professionals	34 (91.7)	3 (8.3)		
**Aware of danger signs in pregnancy**				
No	28 (52.4)	25 (47.6)	44.395	<0.0001*
Yes	120 (94.5)	7 (5.5)		

* Statistically significant

** Likelihood ratio value used

## Discussion

All the respondents recruited for this study participated, giving a response rate of 100%. About 8 out of 10 of the respondents had completed secondary or tertiary education, a value that is higher than the 53.6% obtained for Osun State in 2013 demographic health survey. Osogbo where this study was carried out being the state capital is expected to have more educated people and the health survey was carried out in the whole of Osun State which will include both cities and rural areas with varying educational status. Antenatal care (ANC) is important for a pregnant woman. Initiating ANC early allows early detection and management of any complications, detection of existing diseases and treatment, promotion of health and prevention of disease as well as birth preparedness [9, 16]. One of the most important functions of antenatal care is to offer the woman advice and information about birth preparedness, danger signs of obstetric complication and emergency preparedness. Birth preparedness is a fundamental component of antenatal care whose aim is to reduce any unnecessary delays to seek emergency obstetric care hence improve maternal and foetal outcomes [9, 17]. It is thus impressive when this study revealed more than 90% of respondents who were not primigravida had ANC in their last pregnancy, lesser proportion eventually had their deliveries in health facilities where the services of a skilled birth attendant (SBA) can be guaranteed. This observation also correlates with the National Demographic Health Survey for Osun State [4] where over 90% of women attended ANC, but not all had their deliveries in the health care facilities. Another study in Ethiopia on women who delivered 12 months prior to the study also had 94.4% of their respondents to have attended ANC, but 65% gave birth in health institutions [12]. All these show the importance of continuing education for women on the importance of ensuring SBA take their deliveries. Bleeding per vagina was the most mentioned danger sign during pregnancy and the most mentioned possible complication that may occur during delivery by our respondents. Haemorrhage was also the most known danger sign in pregnancy and delivery in previous studies carried out in Africa [9, 12, 15]. Haemorrhage has been recognized as one of the leading direct cause of maternal mortality in developing countries [18, 19]. Prolonged labour, which is also one of the top five major causes of maternal mortality and topmost cause of morbidity in low-income countries [15], was the second most common possible complications mentioned by our respondents.

This study further showed over 90% of respondents to have identified a place of delivery for the pregnancy they were carrying. In Ethiopia, majority of respondents also said they have identified a place of delivery [12]; similarly, an in-depth interview conducted in a community based survey of among pregnant women and women who delivered in the last 12months in Burkina Faso revealed most women planned to give birth in a health centre [20]. About 8 out of 10 respondents who has identified place of delivery also knew there is presence of skilled birth attendant in the identified place. Beyond identifying place of delivery, availability of skilled birth attendant is paramount as these personnel will ensure “health security”, as reported [20]. A slightly lower proportion was observed in Uganda where about 6 out of the 10 respondents studied were found to have identified a health professional for delivery purpose [15], same was reported in India [8]. The lower proportion reported in these studies may be attributed to location where the studies were carried out; the Ugandan study was carried out in rural area while the India study was carried out in a slum. These two locations usually characteristically have residents to be of low socioeconomic status and low literacy level; however our study was carried out in a city where majority of respondents had completed at least secondary education. Being educated is a positive predictor of planning for birth [8, 15]. Almost ninety percent of the respondents in this study have made transportation arrangement to the place identified for delivery. This is also very important because studies have shown that, although majority of women usually plan to deliver in health care facilities which they must have identified, but many do not make arrangements for transportation, thus making them to deliver outside the health facilities [20]. Making an advance arrangement for transportation reduces or totally eliminates the usual second stage of delay, especially in emergency periods. Advance transport plan should enable a couple know what transport is available at different times of the day, how much it will cost, contact persons or address, alternative mode of transport and more important save money to meet the costs [9]. Over ninety percent of respondents were also found to have saved money towards delivery, had essential baby’s needs ready and had made plans for baby’s special care if required. Only about two-third of the respondents in this study had made arrangement for blood donor, making it the least action respondents have taken with regards to birth preparedness. Previous studies have also reported minimal proportion of respondents making arrangement for blood donor as part of their birth preparedness [1, 9]. This contradicts pregnant women’s knowledge of citing hemorrhage as the commonest danger sign of pregnancy and labour; naturally, one would expect making arrangements for blood donor to be one of the priorities. Further exploratory study may be necessary to investigate why women do not make arrangements for blood donor despite perceiving haemorrhage as a major danger sign. Generally, about 8 out of 10 respondents in this study were well prepared for birth of their babies. Younger age group, being more educated and awareness of the danger signs in pregnancy were factors that that were significantly associated with being well prepared for birth; similar trend has been reported in previous studies [8, 9, 12, 15].

## Conclusion

This study concludes that women in Osogbo metropolis were well prepared for the delivery of their babies with the younger women, educated ones and those that were aware of danger signs being better prepared. Continuous education about the importance of BPACR, most especially at the ANC clinics and in community outreach should be sustained in order to maintain and improve women's preparedness in this area.

### What is known about this topic

- Inadequate or outright lack of birth and emergency preparedness accounts for many maternal deaths in developing countries like Nigeria;
- Birth preparedness and complication readiness (BPACR) is a safe motherhood targeted at ensuring safe delivery and limiting obseric complications;
- In spite of the benefit of BPACR, studies in the developing countries have not shown adequate preparedness.

### What this study adds

- Pregnant women in this study had good knowledge of BPACR and were well prepared for their delivery;
- Younger women and those with high level of education were better prepared than their older and uneducated counterparts;
- Blood donation arrangements were inadequate despite good knowledge of severe hemorrhage as an obstetric complication.

## Competing interests

The authors declare no competing interest.
